# Integrating human biomonitoring exposure data into a primary care morbidity database: a feasibility study

**DOI:** 10.1186/s12940-024-01152-5

**Published:** 2025-01-04

**Authors:** Pieter Jansen, Elly Den Hond, Katleen De Brouwere, Endale Alemayehu Ali, Hamid Yimam Hassen, Ilona Gabaret, Gijs Van Pottelbergh

**Affiliations:** 1 Academic Center for General Practice, KU Leuven, Kapucijnenvoer 7 bus 7001 block h, Leuven, 3000 Belgium; 2 Provincial Institute for Hygiene (PIH), Kronenburgstraat 45, Antwerp, 2000 Belgium; 3 Family Medicine and Population Health, University of Antwerp, Universiteitsplein 1, Wilrijk, 2610 Belgium; 4 Environmental Intelligence Unit, Flemish Institute for Technological Research (VITO), Boeretang 200, Mol, 2400 Belgium; 5 Department of Care Flanders, Simon Bolivarlaan 17, Brussels, 1000 Belgium

**Keywords:** Biological monitoring, Environmental health, Environmental exposure, Electronic health records, Routinely collected health data

## Abstract

**Background:**

The detection of a local per- and polyfluoroalkyl substances (PFAS) pollution hotspot in Zwijndrecht (Belgium) necessitated immediate action to address health concerns of the local community. Several human biomonitoring (HBM) studies were initiated, gathering cross-sectional exposure data from more than 10,000 participants. The linkage of these HBM data with primary care health registries might be a useful new tool in environmental health analysis.

**Aim:**

We assessed the feasibility of linking exposure data from HBM programs to health outcomes from the Intego registry, which collects data from general practitioners’ electronic health records. This feasibility study uses exposure data from one of the completed PFAS HBM studies, which included 796 individuals. We describe the separate datasets, the process of integrating the HBM data into Intego, the analysis plan and the advantages and challenges of using this method.

**Results:**

We established the integration of HBM data into the Intego primary care morbidity database, adhering to stringent privacy regulations and quality standards to ensure result integrity. Because of the modest sample size used in this feasibility study, no conclusions about the impact of PFAS on health endpoints can be drawn. However, with PFAS data from more than 10,000 residents available soon, more robust studies will be possible with this new method.

**Interpretation:**

We introduce a novel approach for assessing the impact of environmental health hazards within primary care settings. The methods outlined here not only pave the way for larger-scale projects but also offer a promising avenue for long-term environmental health monitoring.

**Supplementary Information:**

The online version contains supplementary material available at 10.1186/s12940-024-01152-5.

## Introduction

The impact of our environment on health is an increasingly important topic, given the multifaceted influences of climate change, industrialization and air pollution, among other environmental stressors [1–4]. These challenges significantly affect population well-being, necessitating the development of targeted preventive strategies for individuals vulnerable to adverse health effects from environmental exposures. Achieving such strategies requires a thorough understanding of the nature, timing, and mechanisms underlying these health impacts.

Environmental exposures have complex health implications, influenced by factors such as exposure route, dose, timing, frequency, duration of exposure, genetics and individual lifestyle factors [5]​. Moreover, the diversity of exposure assessment methods, ranging from exposure modelling to environmental monitoring to biomarker measurements in human samples, underscores the importance of cautiously interpreting research findings in this field.

Human biomonitoring (HBM) is defined as the method for assessing human exposure to chemicals or their effects by measuring these chemicals, their metabolites or their reaction products in human samples [6]. It offers a direct measure of internal exposure to environmental pollutants and allows for individualized assessment [7]. Although HBM can sometimes require invasive sampling methods, it remains a powerful approach for assessing internal exposure to environmental pollutants [8].

Over the past few decades, numerous regional HBM studies have been conducted. At the European level, the HBM4EU initiative coordinated these HBM projects with the goal of standardizing procedures across all member states, a project that concluded in June 2022 [9]​. In Flanders, the “Flemish Environment and Health Studies” (FLEHS) have been conducted since 2002 ​[10]​. These studies included four cycles of HBM programs, which involved measuring various pollutants in the population, identifying exposure pathways and determining health outcomes. The fifth cycle is currently underway and is expected to continue until 2027. While many FLEHS and HBM4EU studies have focused on exposure assessment in a population representative sample for Flanders and Europe, other studies have assessed the impact of local pollutants in contaminated regions (hotspots), such as the Veneto cohort studies [11]​​, the Ronneby cohort studies ​[12]​ or the C8 study [13].

HBM studies employ several methods to collect health outcome data. One approach is to have participants complete a health questionnaire, which collects self-reported outcome measures and covariates. However, these self-reported data can be subject to recall bias, making them less than ideal for studies investigating objective health effects. Some HBM studies measure biomarkers of effects in addition to exposure values [14, 15]. Another alternative is to conduct objective measurements, such as weight or blood pressure, during the collection of HBM material [16]. However, measurements of biomarkers of effect and objective parameters are often conducted within a limited subset of the study population.

Integrating HBM data with existing health data, for example from electronic health records (EHRs) of general practitioners (GPs), presents a promising avenue for advancing our understanding of environmental health. This approach can mitigate many limitations of other data collection methods, such as recall bias and the limited availability of objective outcome measures as mentioned before. A wide array of health data is available in EHRs, containing medical information from years before exposure assessments and health outcome evaluations. They continue to collect new medical information after measurements are taken. Additionally, they have a large number of participants creating a larger cohort, and repeated analysis of the exposure makes it possible to create a long-term prospective cohort to follow up on health effects and evolution of the exposure concentration, contributing to evidence for possible causality in any associations that might be found.

There remains a notable gap in research concerning environmental health effects in primary care settings. Primary care often serves as the initial point of contact for individuals experiencing early signs of health issues, including environmental health problems. As the “coordinator of care”, a GP aims to integrate all medical information of patients into their EHR. Integrating GP’s EHRs into environmental health research thus offers a valuable opportunity to enhance our understanding of these health effects at the community level.

However, several issues might hinder this linkage ​[17]​. Both HBM and EHR data contain sensitive personal health information. Ensuring the privacy and confidentiality of these data during the linking process can be complex. Additionally, HBM data and EHRs may not be directly compatible. They might be collected in different formats, use different units of measurement, or categorize data differently. Finally, the process of linking large datasets can be technically demanding, requiring sophisticated software and hardware, as well as expertise in data management and analysis. In this study, we aimed to assess the technical feasibility of integrating HBM data with medical data from GPs’ EHRs.

## Methods

### PFAS human biomonitoring study

After the detection of elevated perfluorooctane sulfonic acid (PFOS) levels in soil and groundwater near a chemical plant in Antwerp, the Flemish government commissioned a population study to assess the extent of PFOS and other per- and polyfluoroalkyl substances (PFAS) exposure within the local population. Residents living within a 3 km radius of the pollution hotspot were invited to undergo blood testing for 16 different PFAS compounds. This study was based on convenience sampling since it relied on volunteers. Ethical approval was granted by the University of Antwerp’s ethical committee on 12/07/2021 (registration number: B3002021000126), and informed consent was obtained from all participants.

In the summer of 2021, 796 residents participated in this project. The study provided descriptive statistics for the 16 PFAS compounds and demonstrated that participants’ PFAS serum levels exceeded background values and health-based guidance values. HBM-I and HBM-II values as described by the German HBM commission were chosen as guidance values used for feedback to participants [18]. Furthermore, the study provided information on the main sources of exposure by analysing the relationships between internal PFAS levels and various environmental and behavioral factors collected via a self-assessed questionnaire. Because the information collected in this questionnaire was not coded electronically like the PFAS measurements, it could not be sent to the GPs’ EHRs and thus could not be used in this specific project. Additionally, we didn’t want to overload the EHR with non-medical information that was collected in the questionnaire (e.g. whether a participant grew vegetables in their backyard). However, the questionnaire and other details of the original exposure assessment study were described by Colles et al. [19]. Participants were given the option to consent to send their PFAS blood test results to their GPs to facilitate individual follow-up. The serum PFAS results were first disseminated to participants’ GPs via post. Only in the second stage, after technical preparation of the data export, the results were delivered electronically and correctly coded to the GP. This process of data integration will be explained further in this article.

The GPs of the affected region received their patients’ results and an information brochure about the possible health effects of PFAS, as well as advice on what to say to their patients about these effects and how to minimize further exposure to PFAS. Patients themselves also received their own results, together with information about guidance values and what they mean. They were referred to an environmental health specialist of the region or their GP if they had any questions regarding their results.

### Intego database

The Intego database serves as a primary care morbidity registry, aggregating health and demographic data extracted from the EHRs of GPs. Stringent measures are in place to ensure the anonymity of medical data. An array of data types can be analysed within the Intego database, including but not limited to age, gender (as registered on the patient’s electronic ID card), postal code, coded diagnoses, prescribed medications, vital signs, and laboratory results. These data are extracted and pseudonymized weekly and can be accessed by authorized members of the Intego team for specific research purposes. To become a registrar for Intego, GP practices must meet specific quality criteria to ensure a high quality of the extracted medical data. A detailed explanation of the data extraction methods, data flow and quality criteria for registrars within Intego are available elsewhere ​[20]​. Only GPs that use CareConnect EHR software, which is used by about 50% of GPs in Flanders, can participate in the Intego project.

As of March 2024, the Intego database contains data from 1,272,166 patients spread across Flanders, Belgium. GPs are constantly being recruited, while some GPs simultaneously leave our network. This creates a dynamic cohort that changes over time.

### Data integration

To integrate the HBM data into the Intego database, we opted to transmit the HBM results directly to the EHR of the participant’s GP. Before we sent the results, each PFAS compound received a designated lab test code, which was also coded into the EHR software. Upon arrival, the PFAS values were thus uniformly coded as lab results within the EHR, enabling automatic extraction by Intego, similar to any other laboratory finding.

A specialized macro was developed to convert the HBM database into .lab files (containing the PFAS result linked to the participant’s social security number) and .adr files (containing the unique RIZIV code of the participant’s GP, which is assigned to every doctor working in Belgium). These files were subsequently dispatched to the participant’s EHR via the eHealth Box utilizing the “Unified Messaging” module (UM module). This method is commonly employed for secure medical data transmission, including communication between GPs and hospitals or laboratories.

### Selection of health endpoints

Given the vast amount of health data within Intego, a selection of health endpoints potentially influenced by PFAS was essential. This selection process was informed by a nonsystematic literature review, drawing from the “Agency for Toxic Substances and Disease Registry” (ATSDR) toxicological profile and several additional cohort studies from other PFAS hotspots in Europe, listing potentially relevant health endpoints for which significant associations with PFAS in serum were found [21–25]​​. Furthermore, the chosen outcomes needed to be readily accessible within the Intego database. As Intego is a primary care morbidity database, data pertaining to mortality or hospitalization were not available. Additionally, for malignancies, a more comprehensive database exists in Belgium, namely the Belgian Cancer Registry [26]. For a full list of selected endpoints, see Table 1. For this study, we selected more prevalent outcomes from this list to assess the feasibility of our methods. Hypertension was used as a test case for chronic binary outcomes. Alanine transaminase (ALT), a liver enzyme, was used as a test case for continuous outcomes.

**Table 1:** List of selected health endpoints that were deemed relevant for analysis and are available in Intego

Category	Biomarker of effect or health endpoint	Data type
**Cardiovascular**	Hypertension	Binary – chronic
	Ischemic events	Binary – acute
	Systolic blood pressure	Continuous
	Diastolic blood pressure	Continuous
**Kidney function**	Renal insufficiency	Binary – chronic
	Creatinine/eGFR	Continuous
**Respiratory**	Chronic bronchitis	Binary – chronic
	Asthma	Binary – chronic
	Shortness of breath	Ordinal
**Hormonal system**	Thyroid disease	Binary – chronic
	Thyroid stimulating hormone (TSH)	Continuous
**Gastro-intestinal**	Ulcerative colitis	Binary – chronic
**Pregnancy**	Gestational hypertension	Binary – acute
**Musculoskeletal**	Arthrosis	Binary – chronic
**Liver function**	Alanine transaminase (ALT)	Continuous
	Aspartate transferase (AST)	Continuous
	Gamma-glutamyl transferase (GGT)	Continuous
	Bilirubin	Continuous
**Fat metabolism**	Total cholesterol	Continuous
	LDL-cholesterol	Continuous
	HDL-cholesterol	Continuous
	Triglycerides	Continuous

Subsequently, we identified the covariates associated with each selected outcome and ascertained their availability within the Intego database using a non-systematic literature search and the clinical experience of the Intego team. In this preliminary study, we used gender, age, BMI, and smoking status as covariates. Owing to the limited sample size, we excluded some less prevalent covariates, such as problematic alcohol use and stress disorders, which were originally considered for ALT and hypertension.

Additionally, a “case definition” was delineated in Intego for all outcomes and covariates, using different coded clinical values such as International Classification of Primary Care (ICPC) codes ​[27]​. For example, renal insufficiency could be defined by the ICPC code for renal insufficiency or by identifying patients with an estimated glomerular filtration rate (eGFR) less than 60 ml/min/1.73m² in multiple measurements over several months. A full list of case definitions for the selected outcomes is available in supplementary Table 1 (S1).

### Statistical analysis

#### Handling of exposure values

Following the integration of PFAS values into the Intego database, our next step was to devise a comprehensive statistical analysis plan. For the exposure values, namely the PFAS serum concentrations, the data can be classified as follows:

- Below the limit of detection (< LOD), meaning the concentration was too low to be detected with current techniques.
- Below the limit of quantification (< LOQ), meaning that PFAS was detected, but at such low concentrations that quantification could not be performed.
- Quantifiable concentration in µg/l.

For data < LOQ, a posttreatment is necessary to include these data in the analysis. Values < LOQ were imputed via simple random imputation techniques if at least 30% of observations in a given biomarker were above the LOQ, in accordance with the method used in HBM4EU [28]​. First, a censored log-normal distribution was fitted through the values above the LOQ. This resulted in the estimation of the mean and standard deviation of the log-normal distribution of all measurements (below and above the LOQ). Values were then randomly imputed for the measurements below the LOQ, drawn between 0 and the limit from the log-normal distribution with the estimated mean and standard deviation. For single-pollutant analysis, linear (L) and the sum of linear + branched (L + B) forms of several PFAS were used. For the mixture models, only the linear forms were used since including both in the model would mean incorporating the linear forms twice in the same model.

#### Data analysis

We categorized the outcomes into different data types: continuous, binary-acute, binary-chronic, or categorical, and devised specific analytical methods for each type. Note that the exposure variables, PFAS serum concentrations, are always continuous. In this feasibility study, we examined the liver value ALT and hypertension as test outcomes as discussed previously.

For ALT (a continuous outcome), we used multiple linear regression analysis.

For hypertension (a chronic binary outcome), we used binary logistic regression analysis.

Moreover, we performed multipollutant mixture analysis to quantify the impact of the PFAS mixture on health outcomes and discern the individual contributions of different PFAS compounds. To achieve this, we adopted the “weighted quantile sum” (WQS) method. The study sample was randomly split into a training set (40%) and a validation set (60%). Using the training set, each chemical was scored into quartiles, and total quantile scores were computed for individual participants. Empirical weights for each PFAS compound in the mixture were estimated by bootstrapping, a statistical resampling procedure. These weights were then used to generate WQS scores representing the overall mixture. To accommodate bidirectional associations (both positive and negative), we conducted quantile-based g-computation, estimating the parameters of a marginal structural model rather than a standard regression model. To assess result consistency and account for potential nonlinear associations between PFAS compounds and health outcomes, we conducted additional mixture analysis using Bayesian kernel machine regression (BKMR) with the ‘BKMR’ package in R. This approach facilitates the identification of uncertain exposure‒outcome relationships, whether linear or nonlinear, through nonparametric methods (kernel function) and subsequently evaluates exposure mixtures. Furthermore, BKMR aids in identifying potential interactions between PFAS compounds.

## Results

In total, 291 patients had at least 1 PFAS compound quantified in their EHR, which was 36.6% of all participants in the HBM study. The main reason for this relatively low number is that not all participants are part of the Intego database: not every GP in the region can participate in the Intego project (e.g. because they use different software or do not meet the quality criteria for registrars as mentioned above) or is willing to participate. Only 29 patients (3.6%) did not give consent to send their HBM results to their GP. As a result of matching all cases with covariates, the sample size was reduced further because of missing data for some of these factors. BMI and smoking status, for example, were not detected in the EHR for every patient. For future studies, imputation techniques will be used to correct for these missing data ​[29]​. Table 2 shows the descriptive analysis for each PFAS.

**Table 2:** Descriptive analysis for the continuous outcome, ALT, and binary outcome, hypertension. L = linear form, B = branched form. L + B = sum of linear and branched forms

PFAS compound	Hypertension	ALT
	Measured count (Detected%, < LOQ%)	
**PFOS (L + B)**^a^	75 (100, 0)	69 (100, 0)
**PFOS (L)**^a^	68 (100, 0)	67 (93, 0)
**PFOA (L + B)**^a^	68 (100, 0)	62 (100, 0)
**PFOA (L)**^a^	68 (100, 0)	62 (100,0)
**PFHxS (L + B)**^a^	68 (100, 0)	62 (100, 0)
**PFHxS (L)**^a^	68 (100, 0)	62 (100, 0)
**PFNA**^a^	68 (100, 0)	62 (100, 0)
**PFHpS**^b^	68 (84, 10)	62 (84, 10)
**PFDA**^b^	68 (94, 7)	62 (94, 5)
**PFUnA**^b^	68 (84, 37)	62 (84, 37)
**PFBA**^b^	68 (81, 69)	62 (81, 67)
**PFHpA**^b^	68 (81, 54)	62 (82, 55)
**PFDoA**^c^	68 (79, 79)	62 (79, 79)
**PFBS**^c^	68 (79, 78)	62 (79, 79)
**PFPeA**^c^	68 (79, 78)	62 (79, 79)
**PFHxA**^c^	68 (79, 78)	62 (79, 79)

^a^Analyses are possible

^b^Analyses are possible with the use of imputation techniques for the exposure value

^c^no analysis is possible because too few quantified exposure values are available

For continuous data, we chose the liver value ALT as the outcome in this first analysis. For binary data, hypertension was selected for testing first. We listed the number of cases we could match with covariates and thus use in the analysis for all PFAS compounds. We also listed the percentage of participants where PFAS were detected (detected%), as well as the percentage of values that were below the LOQ. We were able to perform single-pollutant analyses for 12 compounds, 5 of which needed imputation techniques for the exposure data as described earlier. Furthermore, we were able to perform mixture analysis with the four most prevalent PFAS compounds (PFOS, PFOA, PFHxS and PFNA). However, because of the small sample size, no conclusions can be drawn from these data.

Our multiple regression models revealed significant and clinically logical associations between covariates and health outcomes. For example, patients with a higher BMI had a significantly greater chance of having a hypertension diagnosis. The fact that we find these plausible associations serves as an internal validation of our method.

## Discussion

**Fig. 1:**
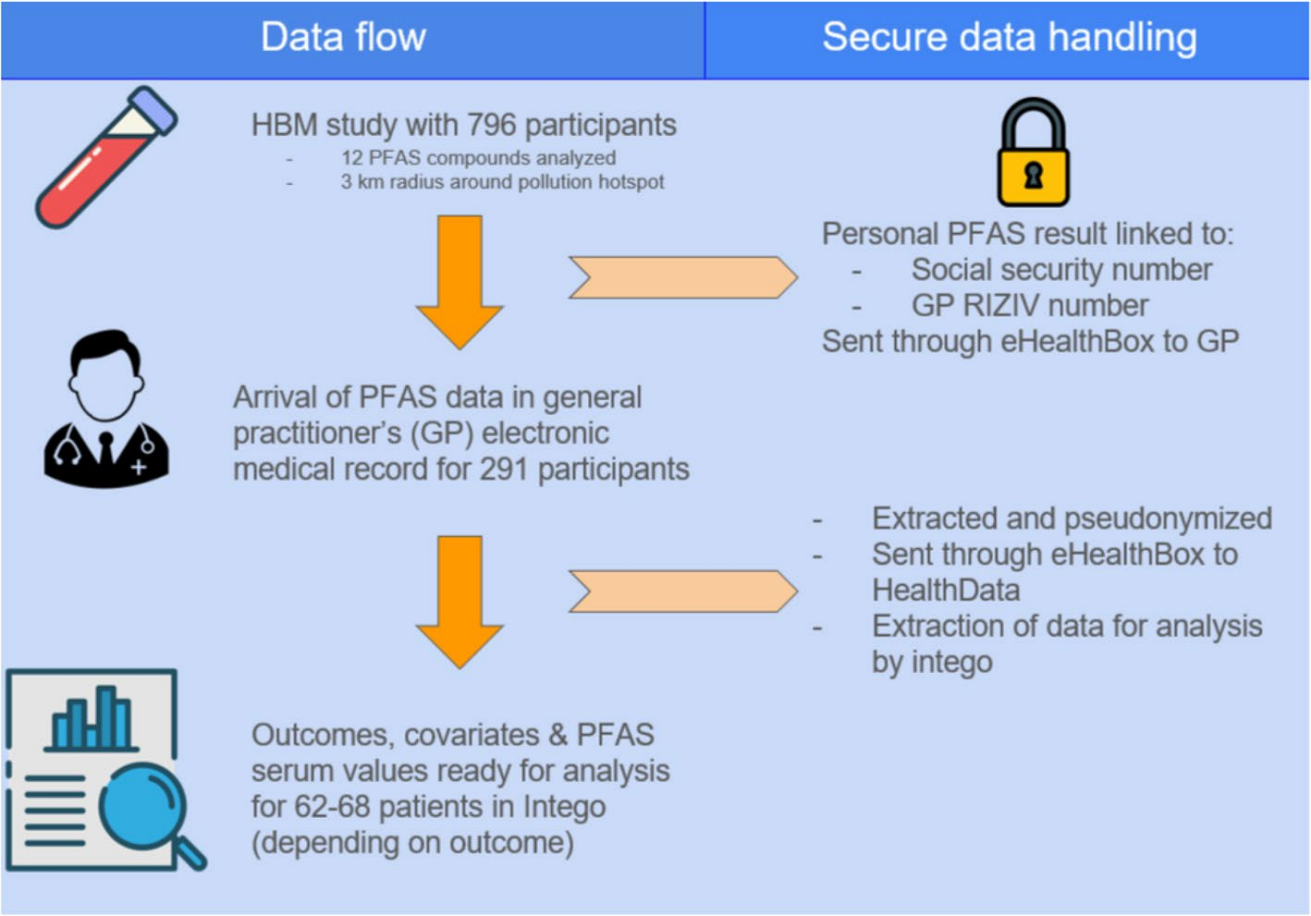
Summary of data flow and data security measures taken at every step

In this study, we explored a new method to investigate the health effects of pollutants in the general population or in specific contaminated areas. We successfully integrated biomarkers of exposure, i.e. serum PFAS values in a population close to a PFAS production site, into Intego, a primary care morbidity database that gathers data from the EHRs of GPs. Figure 1 shows a summary of the data flow and the measures for secure data handling. It should be clear that this study was not designed to draw conclusions about the health impacts of PFAS on the local population. This study’s main objective was to develop the methodology of this novel approach and list its strength and limitations, with the aim of finding ways to improve upon these methods and applying it to larger cohorts in the future.

### Strengths and opportunities

Our method offers several advantages. Its automated data collection from GPs’ EHRs reduces the need for additional data gathering alongside HBM studies, provided that proper coding and consent procedures are adhered to. Moreover, the flexibility to conduct repeated data collection and analysis at chosen intervals enables longitudinal monitoring of affected populations. To the best of our knowledge, this is the first study to establish a direct connection between HBM and primary care health data from EHRs. This new approach helps bridge a gap in environmental health research within primary care that has not been addressed before.

Currently, a larger HBM study is underway in which PFAS levels are measured in more than 9,000 participants in the Zwijndrecht region. This presents a significant opportunity to apply our methods to a larger sample, enabling more robust analyses and addressing the local population’s concerns about the health impact of PFAS. We aspire to extend this methodology to other hotspot cases, including additional PFAS studies and exposure hotspots related to other chemicals (such as metals). We also aim to conduct larger-scale investigations into internal pollution levels among the general Flemish population.

### Challenges and future recommendations

We had to overcome several important challenges when going through the process of data integration, analysis and interpretation. Having learned from these challenges, we propose some recommendations for future studies to ensure a high quality of the integrated data.

#### HBM study setup

First, when setting up HBM projects, informed consent must be given by the participant to send their test results to their GP using their social security number, in order to link them to the data in Intego. Without this, no integration is possible. As mentioned earlier, the majority of patients (96.4%) had no problem with this. Furthermore, to increase the validity of analyses, only labs accredited to analyse the pollutant in question should be used when setting up HBM studies.

Additionally, we noticed that no patients with increased reimbursement were found in the final pool of participants we used for analysis. In 2022, 19.9% of the Flemish population had increased reimbursement for healthcare. This points to severe underrepresentation in our sample. If we look at the data from the HBM study from which we used exposure data, there was also an underrepresentation of people from lower socioeconomic classes, but not as severe as in the final linked dataset. This could be because the sample size was too small or because of the sampling method, since we relied on volunteers in the community to have their blood taken, which may have resulted in selection bias towards a population from a higher social class. Other possibilities are that people with increased reimbursement have fewer covariates coded into their EHR, for example because they visit their GP less often. This merits attention in future studies: people of every socioeconomic status should be included in HBM projects, with extra attention given to those with low income. With more than 10,000 participants from the ongoing PFAS exposure study ready to link with the Intego dataset, this could reflect a more representative sample for the local population.

#### GP data quality

The quality of data registered in the EHR of GPs should be sufficiently high. GPs should be aware of the importance of structurally coded data, not only for research purposes, but also for adequate medical care on individual and community level. A primary care morbidity database should also include as many GPs as possible to create a representative sample of the population, but without losing quality of data registration. This requires educating GPs about how and why to code correctly in their EHRs.

#### Data integration

For sending the data to the EHR, uniform terminology and coding for lab results across all laboratories and EHR software programs are imperative, ideally accompanied by a standardized structure for messaging lab results to each software package. Presently, lab results may not be uniformly integrated into every EHR software package in Flanders. However, given Intego’s exclusive use of CareConnect software, this discrepancy is of lesser concern for this particular study setup.

Privacy-proof methods of data sharing should be used. In Belgium, such a system already exists and was used for the sharing of HBM results with the participant’s GP. For the extraction of information from EHRs to the Intego database, we refer to the Intego protocol paper [20]. In the future, linkage of our integrated dataset to other databases can help improve data quality by adding information that is not currently available in Intego, for example other environmental factors.

#### Data analysis

When performing analyses with our data, the incidence or prevalence of some endpoints in Intego may be too low to allow a valid statistical analysis. We can overcome this problem by enlarging the investigated time interval, e.g. selection of myocardial infarctions in a period of 5 years instead of only 1 year. However, this assumes that the investigated exposure is an indicator of long-term exposure. Additionally, we can also group several endpoints together to increase their prevalence or incidence. For example, when the count for myocardial infarction is too low, we can group it together with other atherosclerotic diseases. This way, more outcomes will be detected and analysis becomes possible. Obviously, we will be as specific as possible and only group endpoints based on clinical relevance. If grouping is done, this will be stated clearly in the methods section of any future study. An example of grouping can be seen in Table 3. In the future, applying this integration method to other databases (such as hospital registers) may lead to higher prevalence or incidence rates of less common outcomes. For example, the incidence of cancer is likely greater in secondary care institutions than in primary care.

**Table 3:** An example of grouping several similar endpoints together in different steps

Disease (ICPC-code)	Group 1	Group 2	Group 3
**K74: Ischemic heart disease with angina**	Ischemic heart disease	Ischemic cardiovascular disease	Cardiovascular disease
**K75: Acute myocardial infarction**			
**K76: Ischemic heart disease without angina**			
**K89: Temporary cerebral ischemia**	Ischemic cerebrovascular disease		
**K90: Stroke**			
**K92: Atherosclerosis/Disease of peripheral arteries**	Peripheral ischemic disease		
**K77: Heart failure**	Heart failure	Heart failure	

#### Interpretation of data analysis

While interpreting the results, several considerations merit attention.

First, several general interpretational issues should be considered when using this method. For example, certain health parameters may exhibit biases. The frequency of measuring kidney function (eGFR) might vary among patients, for example, potentially skewing the results, since patients with renal insufficiency have their eGFR measured more frequently. Additionally, there may be a slight underestimation of disease prevalence or incidence due to underregistration among GPs. However, Intego’s stringent quality criteria for registrars mitigate this concern to a considerable extent. These interpretation challenges can be partially addressed through internal validation of the models, such as assessing the effects of known covariates on outcomes.

Moreover, causal inferences from associations cannot be conclusively drawn from a single cross-sectional analysis. However, the ability for repeated analysis at multiple timepoints can strengthen the evidence for causal relationships. As mentioned before, Intego is a dynamic cohort, with patients leaving and entering our network constantly, meaning that we will lose a certain percentage of patients with HBM values in long-term follow-up. The exact number we will lose because of this will only become clear in future studies. Lastly, the Intego database cannot account for all covariates. Other environmental pollutants or certain lifestyle factors, for example, are not coded in EHRs. This can possibly be overcome in the future by linkage with other databases. This linkage can be facilitated by using the “FAIR Environmental and Health Registry” or “FAIREHR”, which can provide an infrastructure for linking other relevant health and/or environmental data to Intego [30].

## Conclusion

The integration of HBM data into Intego, a primary care morbidity database, required a collaborative effort between researchers in the field of HBM, statisticians, and clinical researchers of Intego. PFAS serum values were successfully integrated into the Intego database, which contains routinely collected medical data. Our method shows that it is feasible to link HBM data to electronic health records.

Although no significant associations were identified in the current dataset, the anticipated influx of more PFAS data into EHRs holds promise for conducting robust analyses in the near future, thereby contributing to the body of evidence concerning the health effects of PFAS. The new method outlined in this study can be swiftly applied to address emerging pollution hotspots, be it for PFAS or other pollutants. Furthermore, the possibility of long-term follow-up in the Intego database creates new opportunities in environmental health research, facilitating investigations into long-term health effects.

## Supplementary Information

Supplementary Material 1.

## Data Availability

The data that support the findings of this study are not openly available due to reasons of sensitivity and are available from the corresponding author upon reasonable request. Data are located in the HealthData dataspace, accessible by the Intego team.

## Abbreviations

ALT: Alanine Transaminase
AST: Aspartate Transferase
ATSDR: Agency for Toxic Substances and Disease Registry
BKMR: Bayesian Kernel Machine Regression
BMI: Body mass index
eGFR: Estimated Glomerular Filtration Rate
EHR: Electronic Health Record
FLEHS: Flemish Environment and Health Studies
gGT: Gamma‒Glutamyl Transferase
GP: General practitioner
HBM: Human Biomonitoring
HDL-cholesterol: High-density lipoprotein cholesterol
LDL-cholesterol: Low-density lipoprotein cholesterol
LOD: Limit of detection
LOQ: Limit of quantification
PFAS: Per- and polyfluoroalkyl substances
PFOS: Perfluorooctane sulfonate
PFOA: Perfluorooctanoic acid
PFNA: Perfluorononanoic acid
PFHxS: Perfluorohexane sulfonic acid
PFHxA: Perfluotohexanoic acid
PFDA: Perfluorodecanoic acid
PFBA: Perfluorobutanoic acid
PFPeA: Perfluoropentanoic acid
PFUnA: Perfluoroundecanoic acid
PFDoA: Perfluorododecanoic acid
PFHpS: Perfluoroheptanesulfonic acid
RIZIV: National Institute for Insurance of Sickness and Disability
TSH: Thyroid stimulating hormone
UM Module: Unified Messaging Module
WQS: Weighted Quantile Sum

## References

[1] Dominski FH, Lorenzetti Branco JH, Buonanno G, Stabile L, Gameiro da Silva M, Andrade A. Effects of air pollution on health: a mapping review of systematic reviews and meta-analyses. Environ Res. 2021;201. 10.1016/J.ENVRES.2021.111487. 10.1016/j.envres.2021.111487 34116013

[2] Liu J, Varghese BM, Hansen A, Zhang Y, Driscoll T, Morgan G, Dear K, Gourley M, Capon A, Bi P. Heat exposure and cardiovascular health outcomes: a systematic review and meta-analysis. Lancet Planet Health. 2022;6:e484–95. 10.1016/S2542-5196(22)00117-6. 35709806 10.1016/S2542-5196(22)00117-6

[3] Romanello M, Cdi Napoli, Green C, Kennard H, Lampard P, Scamman D, Walawender M, Ali Z, Ameli N, Ayeb-Karlsson S, Beggs PJ, Belesova K, Berrang Ford L, Bowen K, Cai W, Callaghan M, Campbell-Lendrum D, Chambers J, Cross TJ, van Daalen KR, Dalin C, Dasandi N, Dasgupta S, Davies M, Dominguez-Salas P, Dubrow R, Ebi KL, Eckelman M, Ekins P, Freyberg C, Gasparyan O, Gordon-Strachan G, Graham H, Gunther SH, Hamilton I, Hang Y, Hänninen R, Hartinger S, He K, Heidecke J, Hess JJ, Hsu SC, Jamart L, Jankin S, Jay O, Kelman I, Kiesewetter G, Kinney P, Kniveton D, Kouznetsov R, Larosa F, Lee JKW, Lemke B, Liu Y, Liu Z, Lott M, Lotto Batista M, Lowe R, Odhiambo Sewe M, Martinez-Urtaza J, Maslin M, McAllister L, McMichael C, Mi Z, Milner J, Minor K, Minx JC, Mohajeri N, Momen NC, Moradi-Lakeh M, Morrissey K, Munzert S, Murray KA, Neville T, Nilsson M, Obradovich N, O’Hare MB, Oliveira C, Oreszczyn T, Otto M, Owfi F, Pearman O, Pega F, Pershing A, Rabbaniha M, Rickman J, Robinson EJZ, Rocklöv J, Salas RN, Semenza JC, Sherman JD, Shumake-Guillemot J, Silbert G, Sofiev M, Springmann M, Stowell JD, Tabatabaei M, Taylor J, Thompson R, Tonne C, Treskova M, Trinanes JA, Wagner F, Warnecke L, Whitcombe H, Winning M, Wyns A, Yglesias-González M, Zhang S, Zhang Y, Zhu Q, Gong P, Montgomery H, Costello A. The 2023 report of the Lancet Countdown on health and climate change: the imperative for a health-centred response in a world facing irreversible harms. Lancet. 2023;402:2346–94. 10.1016/S0140-6736(23)01859-7. 37977174 10.1016/S0140-6736(23)01859-7 PMC7616810

[4] Weilnhammer V, Schmid J, Mittermeier I, Schreiber F, Jiang L, Pastuhovic V, Herr C, Heinze S. Extreme weather events in europe and their health consequences - a systematic review. Int J Hyg Environ Health. 2021;233. 10.1016/J.IJHEH.2021.113688. 10.1016/j.ijheh.2021.113688 33530011

[5] Rattray NJW, Deziel NC, Wallach JD, Khan SA, Vasiliou V, Ioannidis JPA, Johnson CH. Beyond genomics: understanding exposotypes through metabolomics. Hum Genomics. 2018;12. 10.1186/S40246-018-0134-X. 10.1186/s40246-018-0134-x PMC5787293 29373992

[6] Human biomonitoring | EFSA [WWW Document]. URL https://www.efsa.europa.eu/en/glossary/human-biomonitoring. Accessed 18 Jun 24.

[7] Human biomonitoring: Facts and figures. Copenhagen: WHO Regional Office for Europe; 2015. [WWW Document].

[8] Zare Jeddi M, Hopf NB, Louro H, Viegas S, Galea KS, Pasanen-Kase R, Santonen T, Mustieles V, Fernandez MF, Verhagen H, Bopp SK, Antignac JP, David A, Mol H, Barouki R, Audouze K, Duca RC, Fantke P, Scheepers P, Ghosh M, Van Nieuwenhuyse A, Lobo Vicente J, Trier X, Rambaud L, Fillol C, Denys S, Conrad A, Kolossa-Gehring M, Paini A, Arnot J, Schulze F, Jones K, Sepai O, Ali I, Brennan L, Benfenati E, Cubadda F, Mantovani A, Bartonova A, Connolly A, Slobodnik J, Bruinen de Bruin Y, van Klaveren J, Palmen N, Dirven H, Husøy T, Thomsen C, Virgolino A, Röösli M, Gant T, von Goetz N, Bessems J. Developing human biomonitoring as a 21st century toolbox within the European exposure science strategy 2020–2030. Environ Int. 2022;168. 10.1016/J.ENVINT.2022.107476. 10.1016/j.envint.2022.107476 36067553

[9] Gilles L, Govarts E, Rambaud L, Vogel N, Castaño A, Esteban López M, Martin R, Koppen L, Remy G, Vrijheid S, Montazeri M, Birks P, Sepai L, Stewart O, Fiddicke L, Loots U, Knudsen I, Kolossa-Gehring LE, Schoeters M, G. HBM4EU combines and harmonises human biomonitoring data across the EU, building on existing capacity - the HBM4EU survey. Int J Hyg Environ Health. 2021;237. 10.1016/J.IJHEH.2021.113809. 10.1016/j.ijheh.2021.113809 PMC8504197 34455198

[10] Schoeters G, Govarts E, Bruckers L, Den Hond E, Nelen V, De Henauw S, Sioen I, Nawrot TS, Plusquin M, Vriens A, Covaci A, Loots I, Morrens B, Coertjens D, Van Larebeke N, De Craemer S, Croes K, Lambrechts N, Colles A, Baeyens W. Three cycles of human biomonitoring in Flanders - Time trends observed in the flemish environment and health study. Int J Hyg Environ Health. 2017;220:36–45. 10.1016/J.IJHEH.2016.11.006. 28160993 10.1016/j.ijheh.2016.11.006

[11] Pitter G, Da Re F, Canova C, Barbieri G, Jeddi MZ, Daprà F, Manea F, Zolin R, Bettega AM, Stopazzolo G, Vittorii S, Zambelli L, Martuzzi M, Mantoan D, Russo F. Serum levels of Perfluoroalkyl Substances (PFAS) in adolescents and young adults exposed to contaminated drinking Water in the Veneto Region, Italy: a cross-sectional study based on a Health Surveillance Program. Environ Health Perspect. 2020;128. 10.1289/EHP5337. 10.1289/EHP5337 PMC7064325 32068468

[12] Xu Y, Nielsen C, Li Y, Hammarstrand S, Andersson EM, Li H, Olsson DS, Engström K, Pineda D, Lindh CH, Fletcher T, Jakobsson K. Serum perfluoroalkyl substances in residents following long-term drinking water contamination from firefighting foam in Ronneby, Sweden. Environ Int. 2021;147. 10.1016/J.ENVINT.2020.106333. 10.1016/j.envint.2020.106333 33360412

[13] Frisbee SJ, Brooks AP, Maher A, Flensborg P, Arnold S, Fletcher T, Steenland K, Shankar A, Knox SS, Pollard C, Halverson JA, Vieira VM, Jin C, Leyden KM, Ducatman AM. The C8 health project: design, methods, and participants. Environ Health Perspect. 2009;117:1873–82. 10.1289/EHP.0800379. 20049206 10.1289/ehp.0800379 PMC2799461

[14] Wu B, Pan Y, Li Z, Wang J, Ji S, Zhao F, Chang X, Qu Y, Zhu, Yuanduo, Xie L, Li Y, Zhang Z, Song H, Hu X, Qiu Y, Zheng X, Zhang W, Yang Y, Gu H, Li F, Cai J, Zhu Y, Cao Z, Ji S, Lv J, Dai Y, Shi J. X. Serum per- and polyfluoroalkyl substances and abnormal lipid metabolism: A nationally representative cross-sectional study. Environ Int. 2023;172. 10.1016/J.ENVINT.2023.107779. 10.1016/j.envint.2023.107779 36746113

[15] Zheng L, Wang Z, Yang R, Chen W, Zhang J, Li R, Lv W, Lin B, Luo J. The interference between effects of PFAS exposure on thyroid hormone disorders and cholesterol levels: an NHANES analysis. Environ Sci Pollut Res Int. 2023;30:90949–59. 10.1007/S11356-023-28739-8. 37468783 10.1007/s11356-023-28739-8

[16] Qu Y, Lv Y, Ji S, Ding L, Zhao F, Zhu Y, Zhang W, Hu X, Lu Y, Li Y, Zhang X, Zhang M, Yang Y, Li C, Zhang, Miao, Li Z, Chen C, Zheng L, Gu H, Zhu H, Sun Q, Cai J, Song S, Ying B, Lin S, Cao Z, Liang D, Ji JS, Ryan PB, Barr DB, Shi X. Effect of exposures to mixtures of lead and various metals on hypertension, pre-hypertension, and blood pressure: a cross-sectional study from the China National Human Biomonitoring. Environ Pollut. 2022;299. 10.1016/J.ENVPOL.2022.118864. 10.1016/j.envpol.2022.118864 35063540

[17] Chisholm RL, Denny J, Fridsma D, Ohno–Machado L. Opportunities and Challenges related to the use of Electronic Health Records data for research. 2015

[18] Schümann M, Lilienthal H, Hölzer J. Human biomonitoring (HBM)-II values for perfluorooctanoic acid (PFOA) and perfluorooctane sulfonic acid (PFOS) - description, derivation and discussion. Regul Toxicol Pharmacol. 2021;121:104868. 10.1016/J.YRTPH.2021.104868. 33484797 10.1016/j.yrtph.2021.104868

[19] Colles A, Brouwere K, De, Decker A, De, Gabaret I, Hond E, Den, Maele H, Van De, Rooy L, Van, Bautmans B. PFAS in serum of residents living in the neighborhood of a major PFAS manufacturer plant in Belgium. ISEE Conference Abstracts 2022. 2022. 10.1289/ISEE.2022.O-SY-019.

[20] Delvaux N, Aertgeerts B, Van Bussel JCH, Goderis G, Vaes B, Vermandere M. Health Data for Research through a nationwide privacy-proof system in Belgium: design and implementation. JMIR Med Inf. 2018;6. 10.2196/11428. 10.2196/11428 PMC6300317 30455164

[21] ATSDR. Agency for toxic substances and Disease RegistryToxicological Profile for Perfluoroalkyls. Agency for toxic substances and Disease Registry. Atsdr 24. [WWW Document]. 2021.

[22] Canova C, Barbieri G, Zare Jeddi M, Gion M, Fabricio A, Daprà F, Russo F, Fletcher T, Pitter G. Associations between perfluoroalkyl substances and lipid profile in a highly exposed young adult population in the Veneto Region. Environ Int. 2020;145. 10.1016/J.ENVINT.2020.106117. 10.1016/j.envint.2020.106117 32971418

[23] Gallo E, Barbiellini Amidei C, Barbieri G, Fabricio ASC, Gion M, Pitter G, Daprà F, Russo F, Gregori D, Fletcher T, Canova C. Perfluoroalkyl substances and thyroid stimulating hormone levels in a highly exposed population in the Veneto Region. Environ Res. 2022;203. 10.1016/J.ENVRES.2021.111794. 10.1016/j.envres.2021.111794 34358507

[24] Hammarstrand S, Jakobsson K, Andersson E, Xu Y, Li Y, Olovsson M, Andersson EM. Perfluoroalkyl substances (PFAS) in drinking water and risk for polycystic ovarian syndrome, uterine leiomyoma, and endometriosis: a Swedish cohort study. Environ Int. 2021;157. 10.1016/J.ENVINT.2021.106819. 10.1016/j.envint.2021.106819 34391986

[25] Pitter G, Zare Jeddi M, Barbieri G, Gion M, Fabricio ASC, Daprà F, Russo F, Fletcher T, Canova C. Perfluoroalkyl substances are associated with elevated blood pressure and hypertension in highly exposed young adults. Environ Health. 2020;19. 10.1186/S12940-020-00656-0. 10.1186/s12940-020-00656-0 PMC7507812 32958007

[26] Kankerregister. Retrieved April. 3rd 2024, from https://kankerregister.org/nl [WWW Document].

[27] Verbeke M, Schrans D, Deroose S, De Maeseneer J. The International Classification of Primary Care (ICPC-2): an essential tool in the EPR of the GP 809–814. 2006. 17108613

[28] Ottenbros I, Govarts E, Lebret E, Vermeulen R, Schoeters G, Vlaanderen J. Network Analysis to identify communities among multiple exposure biomarkers measured at birth in three flemish General Population samples. Front Public Health. 2021;9. 10.3389/FPUBH.2021.590038. 10.3389/fpubh.2021.590038 PMC7902692 33643986

[29] Mamouris P, Nassiri V, Verbeke G, Janssens A, Vaes B, Molenberghs G. A longitudinal transition imputation model for categorical data applied to alarge registry dataset. StatMed. 2023;42:5405–18. 10.1002/SIM.9919. 10.1002/sim.9919 37752860

[30] Zare Jeddi M, Galea KS, Viegas S, Fantke P, Louro H, Theunis J, Govarts E, Denys S, Fillol C, Rambaud L, Kolossa-Gehring M, Santonen T, van der Voet H, Ghosh M, Costa C, Teixeira JP, Verhagen H, Duca RC, Van Nieuwenhuyse A, Jones K, Sams C, Sepai O, Tranfo G, Bakker M, Palmen N, van Klaveren J, Scheepers PTJ, Paini A, Canova C, von Goetz N, Katsonouri A, Karakitsios S, Sarigiannis DA, Bessems J, Machera K, Harrad S, Hopf NB. FAIR environmental and health registry (FAIREHR)- supporting the science to policy interface and life science research, development and innovation. Front Toxicol. 2023;5. 10.3389/FTOX.2023.1116707. 10.3389/ftox.2023.1116707 PMC10278765 37342468

