# Supplementary material for: Integrating human biomonitoring exposure data into a primary care morbidity database: a feasibility study
Source: Environ Health. 2025 Jan 4;24:1. doi: 10.1186/s12940-024-01152-5 (PMC11700452; doi:10.1186/s12940-024-01152-5)
Supplement: Supplementary file 1 — Supplementary Material 1. [file 12940_2024_1152_MOESM1_ESM.docx]

Supplemental table 1: Case Definitions Intego

Hypertension

- Definition in Intego: Any of the following in medical history (Active) or in Evaluation during consultation in the past year
- K86: Hypertension without end-organ damage
- K87: Hypertension with end-organ damage
- Type of data: binary (Hypertension present yes/no)

Systolic blood pressure

- Definition in Intego: value itself
- Type of data: continuous variable

Diastolic blood pressure

- Definition in Intego: value itself
- Type of data: continuous variable

Ischemic events

- Definition in Intego: any of the following codes in medical history (Active) or in Evaluation during consultation in the 5 years before measurement
- K74: Ischemic heart disease with angina
- K75: Acute myocardial infarction
- K76: Ischemic heart disease without angina
- K89: Temporary cerebral ischemia
- K90: Stroke
- K92: Atherosclerosis/Disease of periferal arteries
- Type of data: Binary (Ischemic disease in the past 5 years yes/no)

ALT

- Definition in Intego: value itself
- Type of data: continuous variable

AST

- Definition in Intego: value itself
- Type of data: continuous variable

GGT

- Definition in Intego: value itself
- Type of data: continuous variable

Billirubin

- Definition in Intego: value itself
- Type of data: continuous variable

Kidney insufficiency and creatinin

- eGFR: Continuous variable, value itself
- Chronic kidney disease:
- Definition in Intego
- Code (U99;N18.9) in medical history (Active) or in Evaluation during consultation in the past year
- OR 2 measurements of eGFR <60 with a minimum of three months in between
- Type of data: Binary (Chronic kidney disease yes/no)

Asthma

- Prevalence
- ICPC-code in medical history (Active) or in Evaluation during consultation in the past 5 years
- R96: Asthma
- Binary value (Diagnosis of asthma yes/no)

Ulcerative colitis

- Definition in Intego, if present in medical history (Active)
- ICPC-code: D94: Chronic enteritis/ulcerative colitis
- Data type: Binary value (UC yes/no)

Thyroid disease

- Definition in Intego
- ICPC-codes, if present in Medical history Active (both) or Inactive – Relevant (T85)
- T85: Hyperthyroidism/thyrotoxicosis
- T86: Hypothyroidism/myxedema
- OR Medication
- L-thyroxine OR Euthyrox (ATC: H03A)
- Strumazol, PTU (ATC: H03B)
- Type of data: binary value (thyroid problem yes/no, if possible this can be divided into hyperthyroidism yes/no or hypothyroidism yes/no)

TSH

- Definition in Intego: value itself
- Data type: continuous variable

Total cholesterol

- Definition in Intego: value itself
- Data type: continuous variable

LDL-C

- Definition in Intego: value itself
- Data type: continuous variable

HDL-C

- Definition in Intego: value itself
- Data type: continuous variable

Triglyceriden

- Definition in Intego: value itself
- Data type: continuous variable

Arthrosis

- Definition in Intego: ICPC-codes
- L84;M47.8 Lumbartrose (lumbar arthrosis)
- L89 Coxartrose  (hip arthrosis)
- L90 Gonartrose (knee arthrosis)
- L91 Other periferal arthroris
- Type of data: Binary (artrhosis in any joint yes/no)

COPD

- Definition in Intego
- ICPC-codes
- R95: Chronic obstructive lung disease
- R79: Chronic bronchitis
  - - - Type of data: Binary (COPD yes/no)

Dyspnea

- Definition in Intego
- R02: Dyspnea
- Type of data: Ordinal data (number per patient)
